# Supplementary material for: Interventions to improve the mental health or mental well-being of migrants and ethnic minority groups in Europe: A scoping review
Source: Glob Ment Health (Camb). 2023 Apr 20;10:e23. doi: 10.1017/gmh.2023.15 (PMC10579672; doi:10.1017/gmh.2023.15)
Supplement: Supplementary file 1 [file S2054425123000158sup.zip › S2054425123000158sup001.pdf]

STUDY PROTOCOL

Interventions to improve the mental health or mental well-being of migrants and ethnic minority groups living in Europe: a scoping review

---

Correspondence regarding this protocol should be addressed to:

Hanne Apers, Hanne.Apers@UAntwerpen.be; Centre for Migration and Intercultural studies, University of Antwerp, Prinsstraat 13, 2000 Antwerp, Belgium

Researchers:

Hanne Apers (Centre for Migration and Intercultural Studies, University of Antwerp)
Lore Van Praag (Centre for Migration and Intercultural Studies, University of Antwerp)
Christiana Nöstlinger (Department of Public Health, Institute of Tropical Medicine, Antwerp)
Charles Agyemang (Academic Medical Centre, University of Amsterdam)

Amendments:

Any amendments to this protocol will be uploaded to the OSF project page, including deviations from previous versions of the protocol.

Open science practices

Preregistration, open material, open data

Funding and conflict of interest statement

This research does not receive any grant from funding agencies in the public, commercial, or not-for-profit sectors. We confirm that there are no conflicts of interest which might influence our work.

Date of submission

2nd of August 2022

State of submission

- Preliminary searches – completed
- Piloting of the study selection process – completed
- Formal screening of search results against eligibility criteria – started
- Extracting of data from selected studies – started

This protocol was prepared in line with the PRISMA-ScR guidelines for scoping reviews (Tricco et al. 2018).

Introduction

Migrant and ethnic minority populations are at greater risk for mental health issues than the general population in Europe and the European Economic Area (EU/EEA) (Carta et al. 2005; Missinne and Bracke 2012; Purgato et al. 2021). Several studies show a higher prevalence of mental disorders such as post-traumatic stress disorder (PTSD), anxiety and depression among these populations, as well as of substance abuse and severe mental illnesses such as psychosis in comparison with the majority population in the countries of residence (Foo et al. 2018; Hynie 2018; Missinne and Bracke 2012; Nosè et al. 2017; Turrini et al. 2017). Migration tends to be a psycho-social process of loss and change, associated with several mental stressors and suffering (Bhugra 2004; Carta et al. 2005; Derr 2017). People who migrate face many challenges prior to and during their migration process, often in the form of human rights violations (Lindert et al. 2017; Priebe, Giacco, and El-Nagib 2016; Purgato et al. 2021). After arrival, the post-migration context appears to be a social determinant affecting migrants' mental health: resettlement stressors such as difficult socio-economic and living circumstances, legal residence procedures, detention procedures, experiences of discrimination and racism, amongst others, may negatively affect their mental health (Lindert et al. 2017; Nosè et al. 2017; Priebe, Giacco, and El-Nagib 2016; Von Werthern et al. 2018). These socio-ecological determinants also affect their offspring and subsequent generations, as similar mental health vulnerabilities are observed among ethnic minorities who are born in European countries (Borrell et al. 2015; Hynie 2018; Ikram 2016; Myers 2009). In addition, intergenerational trauma transmission may affect the mental well-being of migrant descendants (Sangalang and Vang 2017; Silwal et al. 2019). While little data is available on gender difference sregrading these data, some studies point to an increased risk of ill mental health among immigrant women due to Sexual and Gender-Based Violence (SGBV) as a common risk factor faced by many women leaving their native counties to migrate to other countries (La Cascia et al., 2020).

The evidence on the particular causes and circumstances of migrant groups' heightened vulnerabilities to ill mental health, gives reason for specific, targeted interventions on mental health promotion, prevention, and treatment, apart from interventions targeting the general population (Uphoff et al. 2020). Furthermore, migrants and ethnic minorities might experience language, cultural and structural barriers that complicate access to regular mental healthcare (Uphoff et al. 2020). Most of the interventions, however, seem to be directed to the specific subgroup of refugees and asylum seekers, who have specific needs given their specific migration history, distinct legal status and access to health systems (Lebano et al. 2020; Purgato et al. 2021; Uphoff et al. 2020; Nosè et al. 2017). An overview of interventions focusing on these target groups can be found in the Cochrane Library (Uphoff et al. 2020). Additionally, intervention reviews rather seem to focus on those populations diagnosed with a mental health condition and little emphasis is put on the prevention of mental health problems or promotion of mental well-being in those groups without a diagnosis but known to be at increased risk (Purgato et al. 2021). However, preventative strategies and mental health promotion approaches are essential to ensure psychological wellbeing, reduce the mental health burden as well as improve the mental health outcomes of migrant and ethnic minority groups (Foo et al. 2018).

In the current literature, no review can be found on which approaches or factors are effective to prevent or improve the mental health and well-being of migrants and ethnic minority populations in Europe. Given the broad topic that might be addressed through different (study) designs, approaches, and different outcomes observed, we chose a scoping review as methodology guided by the Five steps of the Arksey and O'Malley framework (2005, see below) to summarize available evidence and identify potential knowledge gaps in this indistinct area. This protocol describes the methodological approach, research questions and procedures for the planned scoping review.

Objectives

The review's main objective is to synthesize the available evidence on interventions applied in the EU/EEA +UK to improve migrants' and ethnic minorities' mental health or well-being.

Specific objectives are to identify:

- what interventions are available and their respective outcomes
- intervention mechanisms and culturally adapted delivery strategies
- identify barriers and facilitators to intervention uptake
- give recommendations for policy and practice based on the critical appraisal of the available evidence

Methodology

To conduct our scoping review, we apply Arksey and O'Malley's multistage methodological framework (Arksey and O'Malley 2005), taking into account the refinements made by Levac et al. (Levac, Colquhoun, and O'Brien 2010). The stages are: (1) Clarifying and linking the purpose of the review to the research question, (2) Identifying relevant studies and balancing feasibility with the comprehensiveness of the scoping process, (3) Applying an iterative team approach in the study selection, (4) Charting the data, (5) collating and summarising the results through a qualitative thematic analysis and reporting implications of the findings for policy, practice and research. An optional, additional sixth stage is a consultation round with relevant stakeholders. The systematic data selection is based on the PRISMA extension guidelines for scoping reviews (Tricco et al. 2018).

Step 1: Clarifying and linking the purpose of the review to the research question

Definitions

For the purpose of this review, we applied the following definitions. The term "Migrants and ethnic minorities" describes a heterogeneous group, and there are numerous definitions available to describe this group. For this study, we use the definitions as cited in the glossary of the International Organization for Migration (International Organization for Migration 2019) as a framework. They describe migrants as "*an umbrella term, not defined under international law, reflecting the common lay understanding of a person who moves away from his or her place of usual residence, whether within a country or across an international border, temporarily or permanently, and for a variety of reasons*."

For minorities, they give the following definition "*a group numerically inferior to the rest of the population of a State and/or in a non-dominant position, whose members possess ethnic, religious or linguistic characteristics differing from those of the rest of the population and show, if only implicitly, a sense of solidarity, directed towards preserving their culture, traditions, religion or language*". Application of these definitions includes the group of refugees and asylum seekers, however, we do not include studies if the interventions are solely targeted on refugees and asylum seekers for reasons mentioned in the introduction.

To inform the further scoping process, it is essential to define a clear research question and have a well delineated scope of the subsequent search inquiry (Levac, Colquhoun, and O'Brien 2010). During a first team meeting, the three first authors discussed and decided on the research question based on an **PICo** (**P**opulation, **I**nterest and **Co**ntext) search strategy protocol (Eriksen and Frandsen 2018; Stern, Jordan, and Mcarthur 2014), see table 1. It was agreed that a scoping review methodology was fit for purpose given the broad field of inquiry.

PICo Search Strategy	Inclusion criteria	Exclusion criteria
**Population :** Migrants and Ethnic Minority groups	Migrants as defined by IOM, including second generation migrants. This definition includes asylum seekers and refugees: we do not aim to exclude this group but only include interventions focused on a broader migrant population and not solely targeted on asylum seekers and refugees. When ethnicity/migrant background is included as a control variable and not the focus of the study, we do not include them in the review.	Studies solely focused on refugees and asylum seekers<br>*Rationale*: asylum seekers and refugees have different mental health needs related, and are the scope of another review article in this special Global Mental Health relaunch issue.
	Adults aged +18 years old	Children or adolescents aged -18 years old<br>*Rationale*: children and adolescents may experience different mental health needs and specified intervention approaches.
**Interest:** Interventions to improve mental health and well-being	Studies describing interventions oriented at improving mental health/well-being outcomes; other interventions can be included if they provide a clear link to mental health outcomes	Studies describing interventions without (a link to) outcome measures for mental health or well-being.<br>Studies describing purely medical/pharmacological interventions are excluded.<br>Articles relating to broad policies are excluded from the results, but we may include these articles in the discussion to stimulate debate.
**Context:** EU/EEA + UK and Switzerland	All countries of EU/EEA + UK and Switzerland; if more countries involved in the study (e.g., high income countries), we include the paper but only focus on the European part.	Studies solely focused on countries outside EU/EEA + UK<br>*Rationale:* we only include studies related to the geographical region of Europe to limit the variability in study contexts.

Table 1. Delineating the review question and refining the search strategy based on PICo-approach

The conceptualization of the these terms lead to a clearly articulated scope of inquiry, and enables us to develop the following research question for the review:

> “What is known about interventions applied in the EU/EEA +UK to improve migrants' and ethnic minorities' mental health or well-being?”

Stage 2 – Identifying relevant studies

Eligibility criteria for the search strategy are added to the PICo-criteria, such as year of publication, availability of text and language of publication. We search from 01/01/2000 onwards to include contemporary and relevant intervention studies. We decided to only include peer-review journal articles that are available in full-text and written in English contingent on the available resources. All study designs are included, and we exclude comments, letters to the editor, books and book chapters, conference abstracts and theses. Grey literature is not systematically searched due to quantity of retrievals it may deliver, but a purposive manual search to deepen the discussion of the findings is being performed. Search terms are defined and potential databases discussed, and agreed upon by the review team (i.e. consisting of the first three authors). A few try-out literature searches are conducted by the first author using different databases. Interim results are continuously discussed in team in order to decide on a final, comprehensive search string and agree to conduct the searches in three main relevant bibliographic databases, i.e. PubMed, Web of Science and PsychInfo. Applied search strings can be found in the annex of this protocol.

Stage 3 – Study selection

Studies are selected according to the eligibility criteria (inclusion/exclusion, as listed in the PICo table). All results of the final literature searches are deduplicated using EndNote and are listed by the first author in a Microsoft Excel-file. Authors HA and CN screen all results (title and abstract) independently using a colour code to assign studies to different categories: to include (=green), to exclude (=red), to keep for further full text reading and contingent decision (=orange), and articles to be excluded, but relevant for the discussion section (=blue). Reasons for exclusion after full text reading are being reported and categorized. During several team meetings among the first three authors all results are discussed and a definitive colour is assigned to the individual studies.

Stage 4 – Charting of the data

A data extraction sheet using Microsoft Excel is drafted by the first author and further developed together with the rest of the team, piloting it with several articles. The data extraction sheet compiles the following key characteristics of the full-text articles: author, year of publication, title, country of study, participant characteristics, sample size (if applicable), methodological approach and study design, context of study, phenomenon of interest, theory of change (if applicable), intervention description, used tools to measure outcomes, results and outcomes, barriers, facilitators, recommendations, study limitations. Additional space is foreseen for remarks, if a particular feature of the study was not captured by the previous categories. Reasons for exclusion after full-text reading are recorded. All selected articles are randomly assigned to one of the three first authors to extract data. The categories used in the data extraction sheet form the basis for the next steps, i.e. the synthesis of the results.

Stage 5 – Collating and summarizing the results

A narrative synthesis of the results guided by the objectives and the research question is performed to analyse the relevant thematic, methodological and population-specific characteristics.

Critical Appraisal of Individual Sources of Evidence

Since the aim of this review is to provide an overview of the existing published evidence regardless of methodological quality (Tricco et al. 2018), we do not undertake any form of bias assessment or formal quality rating. However, we critically discuss the findings to reflect on the quality of the available evidence and identify current knowledge gaps.

References

Arksey, Hilary, and Lisa O'Malley. 2005. "Scoping Studies: Towards a Methodological Framework." *International Journal of Social Research Methodology* 8 (1): 19–32. https://doi.org/10.1080/1364557032000119616.

Bhugra, D. 2004. "Migration and Mental Health." *Acta Psychiatr Scand*, no. 109: 243–58.

Borrell, Carme, Laia Palencia, Xavier Bartoll, Umar Ikram, and Davide Malmusi. 2015. "Perceived Discrimination and Health among Immigrants in Europe According to National Integration Policies." *International Journal of Environmental Research and Public Health* 12 (9): 10687–99. https://doi.org/10.3390/ijerph120910687.

Carta, Mauro Giovanni, Mariola Bernal, Maria Carolina Hardoy, Josep Maria Haro-Abad, and "Report on the Mental Health in Europe" working group. 2005. "Migration and Mental Health in Europe (The State of the Mental Health in Europe Working Group: Appendix I)." *Clinical Practice and Epidemiology in Mental Health* 1: 1–16. https://doi.org/10.1186/1745-0179-1-13.

Derr, Amelia Seraphia. 2017. "Mental Health Service Use Among Immigrants in the United States: A Systematic Review." *Psychiatr Serv* 67 (3): 265–74. https://doi.org/10.1176/appi.ps.201500004.Mental.

Eriksen, Mette Brandt, and Tove Faber Frandsen. 2018. "The Impact of PICO as a Search Strategy Tool on Literature Search Quality: A Systematic Review." *Journal of the Medical Library Association* 106 (4): 420–31. https://doi.org/10.5195/jmla.2018.345.

Foo, Shea Q., Wilson W. Tam, Cyrus S. Ho, Bach X. Tran, Long H. Nguyen, Roger S. McIntyre, and Roger C. Ho. 2018. "Prevalence of Depression among Migrants: A Systematic Review and Meta-Analysis." *International Journal of Environmental Research and Public Health* 15 (9). https://doi.org/10.3390/ijerph15091986.

Hynie, Michaela. 2018. "The Social Determinants of Refugee Mental Health in the Post-Migration Context: A Critical Review." *Canadian Journal of Psychiatry* 63 (5): 297–303. https://doi.org/10.1177/0706743717746666.

Ikram, M.U.Z. 2016. *Social Determinants of Ethnic Minority Health in Europe Link to Publication*.

International Organization for Migration. 2019. "INTERNATIONAL Glossary on Migration." www.iom.int.

Lebano, Adele, Sarah Hamed, Hannah Bradby, Alejandro Gil-Salmerón, Estrella Durá-Ferrandis, Jorge Garcés-Ferrer, Fabienne Azzedine, et al. 2020. "Migrants' and Refugees' Health Status and Healthcare in Europe.Pdf." *BMC Public Health* 20 (1039). https://doi.org/https://doi.org/10.1186/s12889-020-08749-8.

Levac, Danielle, Heather Colquhoun, and Kelly K. O'Brien. 2010. "Scoping Studies: Advancing the Methodology." *Implementation Science* 5 (1): 1–9. https://doi.org/10.1186/1748-5908-5-69.

Lindert, Jutta, Meryam Schouler-Ocak, Andreas Heinz, and Stefan Priebe. 2017. "Mental Health , Health Care Utilisation of Migrants in Europe" 9338 (February 2008). https://doi.org/10.1016/S0924-9338(08)70057-9.

Missinne, Sarah, and Piet Bracke. 2012. "Depressive Symptoms among Immigrants and Ethnic Minorities: A Population Based Study in 23 European Countries." *Social Psychiatry and Psychiatric Epidemiology* 47 (1): 97–109. https://doi.org/10.1007/s00127-010-0321-0.

Myers, Hector F. 2009. "Ethnicity- and Socio-Economic Status-Related Stresses in Context: An Integrative Review and Conceptual Model." *Journal of Behavioral Medicine* 32 (1): 9–19.

https://doi.org/10.1007/s10865-008-9181-4.

Nosè, Michela, Francesca Ballette, Irene Bighelli, Giulia Turrini, Marianna Purgato, Wietse Tol, Stefan Priebe, and Corrado Barbui. 2017. “Psychosocial Interventions for Post-Traumatic Stress Disorder in Refugees and Asylum Seekers Resettled in High-Income Countries: Systematic Review and Meta-Analysis.” *PLoS ONE* 12 (2): 1–16. https://doi.org/10.1371/journal.pone.0171030.

Priebe, S, D Giacco, and R El-Nagib. 2016. “WHO Health Evidence Network Synthesis Reports.” *Public Health Aspects of Mental Health Among Migrants and Refugees: A Review of the Evidence on Mental Health Care for Refugees, Asylum Seekers and Irregular Migrants in the WHO European Region*.

Purgato, Marianna, Justin Richards, Eleonora Prina, Ahlke Kip, Lidia Del Piccolo, Giulia Michencigh, Michela Rimondini, et al. 2021. “Efficacy of Physical Activity Interventions on Psychological Outcomes in Refugee, Asylum Seeker and Migrant Populations: A Systematic Review and Meta-Analysis.” *Psychology of Sport and Exercise* 54 (January). https://doi.org/10.1016/j.psychsport.2021.101901.

Sangalang, Cindy C., and Cindy Vang. 2017. “Intergenerational Trauma in Refugee Families: A Systematic Review.” *Journal of Immigrant and Minority Health* 19 (3): 745–54. https://doi.org/10.1007/s10903-016-0499-7.

Silwal, Sanju, Venla Lehti, Roshan Chudal, Auli Suominen, Lars Lien, and Andre Sourander. 2019. “Parental Immigration and Offspring Post-Traumatic Stress Disorder: A Nationwide Population-Based Register Study.” *Journal of Affective Disorders* 249: 294–300. https://doi.org/10.1016/j.jad.2019.02.002.

Stern, Cindy, Zoe Jordan, and Alexa Mcarthur. 2014. “Developing the Review Question and Inclusion Criteria.” *American Journal of Nursing* 114 (4): 53–56. https://doi.org/10.1097/01.NAJ.0000445689.67800.86.

Tricco, Andrea C., Erin Lillie, Wasifa Zarin, Kelly K. O’Brien, Heather Colquhoun, Danielle Levac, David Moher, et al. 2018. “PRISMA Extension for Scoping Reviews (PRISMA-ScR): Checklist and Explanation.” *Annals of Internal Medicine* 169 (7): 467–73. https://doi.org/10.7326/M18-0850.

Turrini, Giulia, Marianna Purgato, Francesca Ballette, Michela Nosè, Giovanni Ostuzzi, and Corrado Barbui. 2017. “Common Mental Disorders in Asylum Seekers and Refugees: Umbrella Review of Prevalence and Intervention Studies.” *International Journal of Mental Health Systems* 11 (1): 1–14. https://doi.org/10.1186/s13033-017-0156-0.

Uphoff, Eleonora, Lindsay Robertson, Baltica Cabieses, Francisco J Villalón, Marianna Purgato, Rachel Churchill, and Corrado Barbui. 2020. “An Overview of Systematic Reviews on Mental Health Promotion, Prevention, and Treatment of Common Mental Disorders for Refugees, Asylum Seekers, and Internally Displaced Persons.” *Cochrane Database of Systematic Reviews* 2020 (9). https://doi.org/10.1002/14651858.CD013458.pub2.

Werthern, M. Von, K. Robjant, Z. Chui, R. Schon, L. Ottisova, C. Mason, and C. Katona. 2018. “The Impact of Immigration Detention on Mental Health: A Systematic Review.” *BMC Psychiatry* 18 (1): 1–19. https://doi.org/10.1186/s12888-018-1945-y.

Annex 1

Development of search string Web of Science

	Keyword	Search String		
#1	Interventions		TOPIC	("Intervention" OR "Measure" OR "approach")
#2	To improve	AND	TOPIC	("improv*" OR "promot*" OR "prevent*" OR "optimiz*" OR "optimis*" OR "supporting")
#3	Mental health	AND	TOPIC	("mental health" OR "well-being" OR "mental health outcomes" OR "psycholog*" OR "psychosoc*")
#4	Migrants and ethnic minorities	AND	TOPIC	("migra*" OR "ethn*" AND "minorit*")
#5	EU/EEA+UK	AND	TOPIC	("European Union" OR "EU" OR "EEA" OR "Belgium" OR "Bulgaria" OR "Cyprus" OR "Denmark" OR "Germany" OR "Estonia" OR "Finland" OR "France" OR "Greece" OR "Hungary" OR "Ireland" OR "Italy" OR "Croatia" OR "Latvia" OR "Lithuania" OR "Luxembourg" OR "Malta" OR "Netherlands" OR "Austria" OR "Poland" OR "Portugal" OR "Romania" OR "Slovenia" OR "Slovakia" OR "Spain" OR "Czech Republic" OR "United Kingdom" OR "Sweden" OR "England" OR "Scotland" OR "Wales" OR "Northern Ireland" OR "Iceland")
	Publication date<br>From 01/01/2000 to 07/06/2022			

((((TS=(("Intervention" OR "Measure" OR "approach"))) AND TS=(("improv*" OR "promot*" OR "prevent*" OR "optimiz*" OR "optimis*" OR "supporting"))) AND TS=(("mental health" OR "well-being" OR "mental health outcomes" OR "psycholog*" OR "psychosoc*"))) AND TS=(("migra*" OR "ethn*" AND "minorit*"))) AND TS=((“European Union” OR “EU” OR “EEA” OR “Belgium” OR “Bulgaria” OR “Cyprus” OR “Denmark” OR “Germany” OR “Estonia” OR “Finland” OR “France” OR “Greece” OR “Hungary” OR “Ireland” OR “Italy” OR “Croatia” OR “Latvia” OR “Lithuania” OR “Luxembourg” OR “Malta” OR “Netherlands” OR “Austria” OR “Poland” OR “Portugal” OR “Romania” OR “Slovenia” OR “Slovakia” OR “Spain” OR “Czech Republic” OR “United Kingdom” OR “Sweden” OR “England” OR “Scotland” OR “Wales” OR “Northern Ireland” OR “Iceland”))

Development of search string PubMed & PsychInfo:

	Keyword	Search String
#1	Interventions	("Intervention" OR "Measure" OR "approach")
#2	To improve	("improv*" OR "promot*" OR "prevent*" OR "optimiz*" OR "optimis*" OR "supporting")
#3	Mental health	("mental health" OR "well-being" OR "mental health outcomes" OR "psycholog*" OR "psychosoc*")
#4	Migrants and ethnic minorities	("migra*" OR "ethn*" AND "minorit*")
#5	EU/EEA+UK	(“European Union” OR “EU” OR “EEA” OR “Belgium” OR “Bulgaria” OR “Cyprus” OR “Denmark” OR “Germany” OR “Estonia” OR “Finland” OR

		“France” OR “Greece” OR “Hungary” OR “Ireland” OR “Italy” OR “Croatia” OR “Latvia” OR “Lithuania” OR “Luxembourg” OR “Malta” OR “Netherlands” OR “Austria” OR “Poland” OR “Portugal” OR “Romania” OR “Slovenia” OR “Slovakia” OR “Spain” OR “Czech Republic” OR “United Kingdom” OR “Sweden” OR “England” OR “Scotland” OR “Wales” OR “Northern Ireland” OR “Iceland”)
	#1 AND # 2 AND # 3 AND #4 and #5	
	+ Filter: 01/01/2000 - 07/06/2022	

("Intervention" OR "Measure" OR "approach") AND ("improv*" OR "promot*" OR "prevent*" OR "optimiz*" OR "optimis*" OR "supporting") AND ("mental health" OR "well-being" OR "mental health outcomes" OR "psycholog*" OR "psychosoc*") AND ("migra*" OR "ethn*" AND "minorit*") AND (“European Union” OR “EU” OR “EEA” OR “Belgium” OR “Bulgaria” OR “Cyprus” OR “Denmark” OR “Germany” OR “Estonia” OR “Finland” OR “France” OR “Greece” OR “Hungary” OR “Ireland” OR “Italy” OR “Croatia” OR “Latvia” OR “Lithuania” OR “Luxembourg” OR “Malta” OR “Netherlands” OR “Austria” OR “Poland” OR “Portugal” OR “Romania” OR “Slovenia” OR “Slovakia” OR “Spain” OR “Czech Republic” OR “United Kingdom” OR “Sweden” OR “England” OR “Scotland” OR “Wales” OR “Northern Ireland” OR “Iceland”)
