# Supplementary material for: Interventions to improve the mental health or mental well-being of migrants and ethnic minority groups in Europe: A scoping review
Source: Glob Ment Health (Camb). 2023 Apr 20;10:e23. doi: 10.1017/gmh.2023.15 (PMC10579672; doi:10.1017/gmh.2023.15)
Supplement: Supplementary file 1 [file S2054425123000158sup.zip › S2054425123000158sup002.pdf]

Annex to the article “Interventions to improve the mental health or mental well-being of migrants and ethnic minority groups in Europe: a scoping review”

Search string applied:

(("Intervention" OR "Measure" OR "approach") AND ("improv*" OR "promot*" OR "prevent*" OR "optimiz*" OR "optimis*" OR "supporting") AND ("mental health" OR "well-being" OR "mental health outcomes" OR "psycholog*" OR "psychosoc*") AND ("migra*" OR "ethn*" AND "minorit*") AND ("European Union" OR "EU" OR "EEA" OR "Belgium" OR "Bulgaria" OR "Cyprus" OR "Denmark" OR "Germany" OR "Estonia" OR "Finland" OR "France" OR "Greece" OR "Hungary" OR "Ireland" OR "Italy" OR "Croatia" OR "Latvia" OR "Lithuania" OR "Luxembourg" OR "Malta" OR "Netherlands" OR "Austria" OR "Poland" OR "Portugal" OR "Romania" OR "Slovenia" OR "Slovakia" OR "Spain" OR "Czech Republic" OR "United Kingdom" OR "Sweden" OR "England" OR "Scotland" OR "Wales" OR "Northern Ireland" OR "Iceland" OR "Norway" OR "Switzerland" OR "Lichtenstein"))

Table: Quantitative outcome measures (selection of quantitative and mixed-methods studies)

**Authors + publication year**	**Quantitative outcomes measurement tools**	**Remarks in text concerning cultural validity/adaptation of tools**
Afuwape et al., 2010<br>(*RCT*)	*Study eligibility:* WHO Mental Health Checklist for Anxiety and Depression<br>GHQ- 28: General Health Questionnaire<br>CSRI, Client Service Receipt Inventory - adapted form<br>SF-36, Short Form-36 (quality of life)<br>GAF, Global Assessment of Functioning<br>LEDS, Life Events and Difficulties Schedule	Not discussed
Chaudhry et al., 2009<br>(*Quantative*, *observational pilot study*)	SRQ, Self-Reporting Questionnaire (Urdu Version)<br>SCAN, Schedule for Clinical Assessment in Neuropsychiatry	Urdu version of SRQ, validated in specific target population
Eylem et al., 2021<br>(*Mixed methods study – RCT with qualitative component*)	Feasibility: SUS, System Usability Scale<br>BSS, Beck’s Suicidal Ideation Scale<br>BHS, Beck Hopelessness Scale<br>PSWQ-PW, Penn State Worry Questionnaire<br>EQ-5D, Euro Quol – 5 dimensions (Quality of life)<br>SASH, Suicide Attempts and Self-Harm<br>LAS, Lowlands Acculturation Scale (adapted)	All scales show internal consistency. BDI and EQ-5D have been validated in Turkish and Dutch populations. The Turkish version of PSWQ was used. Adapted version of LAS internally consistent in Turkish migrant populations.

Gater et al., 2010<br>(*Mixed methods study – RCT with qualitative component*)	HRSD, Hamilton Rating Scale for Depression<br>Verona Service Satisfaction Scale (adapted)<br>Specific scale developed for rating social functioning among Pakistani women	Specific scale developed for rating social functioning among Pakistani women; measures were (previously) translated; all questionnaires were read to participants to involve low literate participants
Hesselink et al., 2012<br>(*Non-ranomized trial study*)	CES-D, Center for Epidemiologic Studies Depression Scale	Previously validated in target group
Jacob, Bhugra & Mann, 2002<br>(*RCT*)	GHQ-12, General Health Questionnaire	GHQ has previously been validated among British Asians. Patients could choose English or Hindi as language for the questionnaire.
Khan et al., 2019<br>(*Mixed-methods feasibility study*)	EPDS, Edinburgh Postnatal Depression Scale<br>CIS-R, Clinical Interview Schedule – Revised<br>EQ-5D, EuroQol (quality of life)<br>DAS, Dyadic adjustment scale<br>MSPSS, Multidimensional scale of perceived social support<br>Verona Service Satisfaction Scale (adapted)	EPDPS was previously validated with the target group; other instruments were previously used with the target group in other studies.
Knifton et al., 2010<br>(*Mixed-methods cross-sectional study*)	Self-developed questionnaire, using similar wording to the national Scottish survey of public attitudes to mental health.	Questionnaire was translated into Chinese, Urdu and Hindi, group workers available
Kocken et al., 2008<br>(*RCT*)	Combined questionnaire with 14 items from BIOPRO, Biographical Problem Inventory List; five items measuring relation between pain and stress; nine items from validated questionnaire on perceived social support, two items from SF36, Short Form Health survey<br>SCL-90, Symptom CheckList (measuring mental health)	Only the unitary index of psychological discomfort of the SCL-90 was presented because of target group; questionnaire was (back) translated by certified interpreters and native speaker
Lovell et al., 2014<br>(*Mixed-methods study, exploratory randomized trial*)	CORE-OM, Clinical Outcomes in Routine Evaluation – Outcome Measure Scale<br>PHQ-9, Patient Health Questionnaire<br>GAD-7, Generalised Anxiety Disorder assessment<br>WSAS, Work and Social Adjustment Scale<br>EQ-5D, EuroQol (quality of life)	Instruments have previously been used in non-English speaking populations. Existing translations for target languages were used and in case of non-availability instruments were translated and adapted as per published guidelines.
Osman et al., 2017<br>(*RCT*)	GHQ-12, General Health Questionnaire<br>PSOC, Parenting Sense of Competence	Both instruments translated according to international guidelines.

Osman et al., 2021<br>(*Mixed methods longitudinal impact study of Osman et al, 2017)*	CBCL 6-18, Child Behaviour Checklist 6-18 years (adapted)<br>GHQ-12, General Health Questionnaire	Both instruments were translated following the five steps of the WHO's process of translation. CBCL adapted for Muslim respondents.
Rabiee et al., 2015<br>(*Mixed methods study*)	Questionnaire evaluating experience in accessing services and perceived changes in health and well-being (developed on basis of literature review)	Two bilingual researchers were trained to administer the questionnaire. Questionnaire was slightly adapted after piloting with the target group.
Reijneveld et al., 2003<br>(*RCT*)	SF-12/36, Short Form<br>Self-constructed items for measuring knowledge on health and disease, and physical activity	All measures were translated to Turkish by certified Turkish interpreters, and translated back by an independent native speaker and piloted among the target group.
Siddiqui et al., 2019<br>(*RCT*)	MADRS-S, Montgomery Asberg Depression Rating Scale<br>HADS, Hospital Anxiety and Depression Scale	MADRS-S has not been validated in target population, but HADS shows good validity
Ünlü-Ince et al., 2013<br>(*RCT*)	CES-D, Center for Epidemiologic Studies Depression Scale<br>HADS, Hospital Anxiety and Depression Scale<br>SCL-90-R, Symptom Checklist-90-Revised (somatization subscale)<br>EQ-5D, EuroQol (quality of life)	Dutch, Turkish and online versions of CES-D have shown good validity and reliability. HADS, SCL-90-R and EQ-5D translations validated by previous studies.
